# Supplementary material for: Hotspots of the stokes rotating circulation in a large marginal sea
Source: Nat Commun. 2022 Apr 25;13:2223. doi: 10.1038/s41467-022-29610-z (PMC9038737; doi:10.1038/s41467-022-29610-z)
Supplement: Supplementary file 1 — Supplementary Information [file 41467_2022_29610_MOESM1_ESM.pdf]

Supplementary Information for Hotspots of the Stokes Rotating Circulation in a Large Marginal Sea

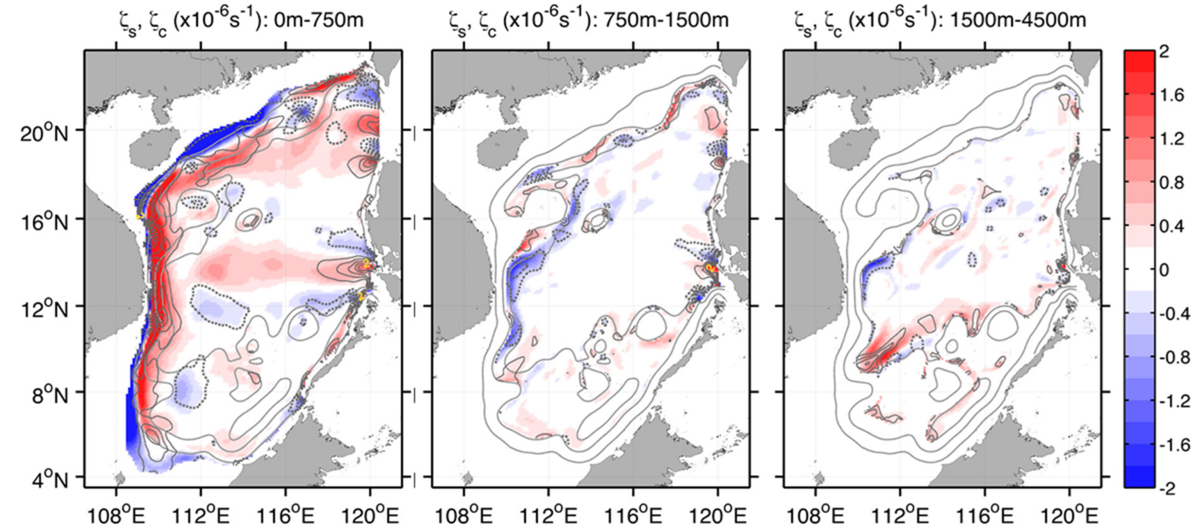

Supplementary Figure 1. Shear (ζ_s) and curvature (ζ_c) vorticity in the SCS. The color contours refer to ζ_s. The solid and dashed contours are positive and negative $\boldsymbol{\zeta_c}$, respectively.

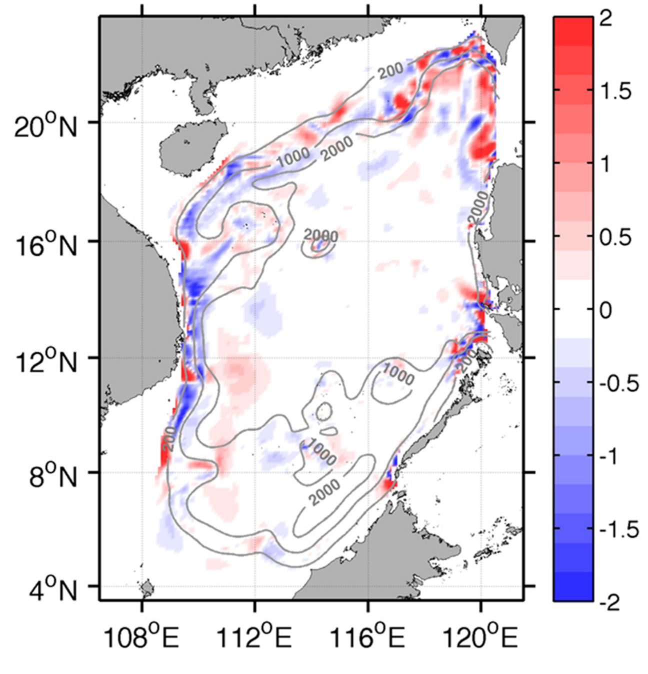

Supplementary Figure 2. Horizontal vorticity advection (10^{-9} m s^{-2}) in the upper layer. The contour lines represent the bathymetry (m).

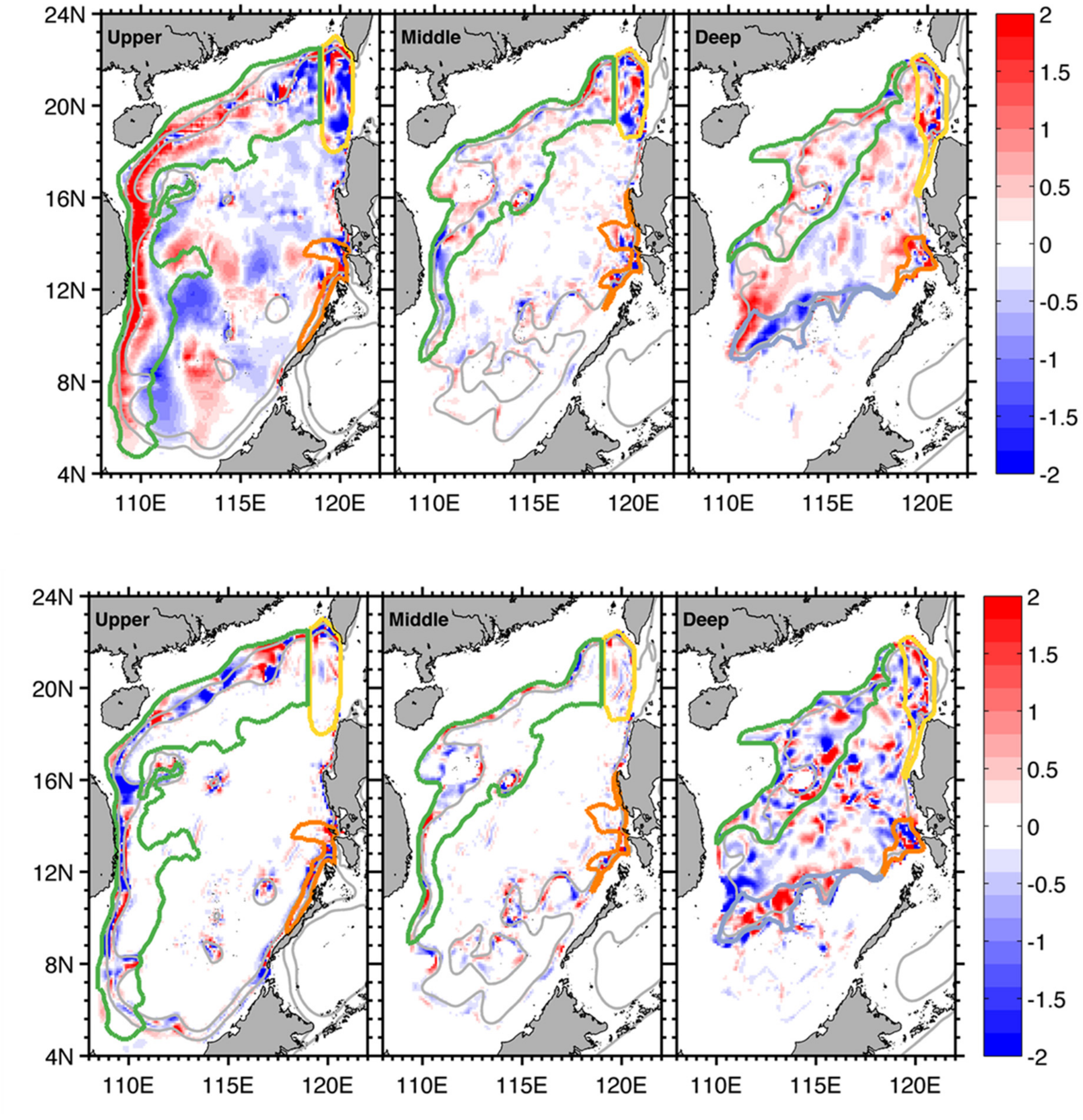

Supplementary Figure 3. Terms of Ω_{cor} and Ω_{pgf} in vorticity equation. Horizontal distribution of the 20-year means of the (a) Ω_{cor} and (b) Ω_{pgf} terms (10^{-9}, m^2 s^{-2}) from Equation (5). The data were averaged in the upper (<750 m), middle (750-1500 m), and deep (>1500 m) layers. The colored circled areas represent the hotspots: Luzon (yellow), SLO (green), Mindoro (purple), and SWB (blue), respectively. The grey contours are the 200 m, 1500 m and 2500 m isobaths in the upper, middle, and deep layers, respectively.
